# 

**Published:** 1886-10

**Authors:** 


### BOOKS RECEIVED.

Reference Handbook of the Medical Sciences, vol. iii.: Wm. Wood & Co., New York.

Handbook of General Therapeutics, vol. v.: Wm. Wood & Co., New York.

A System of Practical Medicine, vol. v.: Lea Brothers & Co., Philadelphia.

The Physicians' Leisure Library Series—six numbers: Geo. S. Davis, Detroit.

A Treatise on Electrolysis: Wm. Wood & Co., New York.

The Nature, Pathology and Treatment of Rheumatism: Wm. Wood & Co., New York.

### OUR ADVERTISERS.

DISSECTING CASES.—The attention of medical students is called to the advertisement of Mr. I. Phillips, the instrument dealer at 13 North Broad street. He has a very large stock of dissecting cases on hand at remarkably low prices. Call and see him. His is the only instrument house in the South.

ACID MANNATE IN CONSTIPATION IN PREGNANCY.—Dr. F. W. Bathrick, Battle Creek, Mich., says: "I used acid mannate with great satisfaction in the case of a young lady in her first pregnancy. She was most obstinately constipated for five months—had taken everything she could hear of. No relief. She was entirely cured with one bottle of acid mannate. Completed her term, and has remained well ever since—now four months."

COMPARATIVE TESTS OF INFANT FOODS.—W. H. Rassman, M. D., Attending Physician Northeastern Dispensary and Late House Surgeon, Maternity Hospital, New York city, under date of August 1, 1886, reports as follows: "Impressed with the importance of the proper feeding of infants, I determined to make as thoroughly as possible a series of comparative clinical tests, both in the Northeastern Dispensary and in private practice. I obtained eleven varieties of food, using of each one or more packages according to the duration of the case. I used as little medicine as possible, and took particular care to have the directions on each package of food carefully followed. Some foods I found absolutely worthless, if not injurious, containing undigested starch and other elements. Others seemed useful in simple mal-nutrition, but were too laxative and irritating in their action to be safe in intestinal derangements. The food, however, which in any and every case fulfilled all requirements was lactated food. In mal-nutrition it was 'a complete substitute for mother's milk,' acting in a way which charmed the mother, and was highly appreciated by the physician. In the exhaustion consequent on summer diarrhœa and entero-colitis, its effect was wonderful, filling out the emaciated body and checking the disease with little or no medicine. In cholera infantum, however, it achieved its greatest triumph, holding the disease in check in a grand manner, and finally restoring fully the lost weight and strength."

